# Identification of microRNA that represses IRS-1 expression in liver

**DOI:** 10.1371/journal.pone.0191553

**Published:** 2018-01-24

**Authors:** Kaoru Ono, Motoyuki Igata, Tatsuya Kondo, Sayaka Kitano, Yuki Takaki, Satoko Hanatani, Masaji Sakaguchi, Rieko Goto, Takafumi Senokuchi, Junji Kawashima, Noboru Furukawa, Hiroyuki Motoshima, Eiichi Araki

**Affiliations:** Department of Metabolic Medicine, Faculty of Life Sciences, Kumamoto University, Kumamoto, Japan; Institut de Pharmacologie Moleculaire et Cellulaire, FRANCE

## Abstract

MicroRNAs (miRNAs) are short, non-coding RNAs that post-transcriptionally regulate gene expression and have been shown to participate in almost every cellular process. Several miRNAs have recently been implicated in glucose metabolism, but the roles of miRNAs in insulin-resistant conditions, such as obesity or type 2 diabetes, are largely unknown. Herein, we focused on miR-222, the expression of which was increased in the livers of high fat/high sucrose diet-fed mice injected with gold thioglucose (G+HFHSD). Overexpression of miR-222 in primary mouse hepatocytes attenuated Akt phosphorylation induced by insulin, indicating that miR-222 negatively regulates insulin signaling. As per *in silico* analysis, miR-222 potentially binds to the 3′ untranslated region (3′ UTR) of the *IRS-1* gene, a key insulin signaling molecule. In fact, IRS-1 protein expression was decreased in the livers of G+HFHSD-fed mice. We further confirmed a direct interaction between miR-222 and the 3′ UTR of *IRS-1* via luciferase assays. Our findings suggest that up-regulation of miR-222 followed by reduction in IRS-1 expression may be a viable mechanism of insulin resistance in the liver.

## Introduction

Insulin resistance is one of the major factors contributing to the development of type 2 diabetes. We have previously investigated the molecular mechanism governing gene expression of key players involved in insulin signaling, including insulin receptor [1,2], insulin receptor substrate 1 (IRS-1) [3,4], and IRS-2 [5]. In 3T3-F442A adipocytes, insulin decreased IRS-1 protein expression without affecting its mRNA levels or promoter activity. Dexamethasone also decreased IRS-1 protein expression without affecting mRNA levels or promoter activity [3]. These results indicated that insulin and dexamethasone down-regulate IRS-1 expression post-transcriptionally. As for insulin-mediated regulation of IRS-1, it seemed to be, at least in part, due to a decrease in the half-life of IRS-1 protein. In cultured hepatocytes, chronic exposure to insulin decreased IRS-2 protein expression concomitantly with a decrease in *Irs-2* mRNA levels. Some nuclear proteins were shown to bind to the insulin response element sequence on the *Irs-2* gene in an insulin-dependent manner. We concluded that insulin decreased IRS-2 expression through suppression of its promoter activity [5]. Subsequently, Nakagawa et al. reported that transcription factor binding to transcription factor enhancer 3 (TFE3) and transcription factor forkhead box class O (FoxO) 1 activated the *IRS-2* promoter and induced *IRS-2* gene transcription [6]. Furthermore, Ide et al. reported that insulin-activated SREBPs repressed *IRS-2* gene transcription by blocking the FoxO/TFE3 complex’s access to the *IRS-2* promoter [7]. These observations suggested that IRS-1 and IRS-2 protein expression are negatively regulated by chronic insulin stimulation or under the state of insulin resistance. However, the regulatory mechanisms, particularly those of IRS-1, are not well understood.

MicroRNAs (miRNAs) are short, non-coding RNAs that bind to the 3′ untranslated regions (3′ UTRs) of target mRNAs and repress their expression by either transcript destabilization, translational inhibition, or both [8–10]. Since the discovery of miRNAs as regulators of developmental timing in *Caenorhabditis elegans*, they have been shown to participate in almost every cellular process investigated, including cell proliferation and differentiation, apoptosis, and metabolic homeostasis [9,11–13]. A recent study of circulating miRNA demonstrated that exosomal miRNAs could act as regulators of metabolism in distant tissues [14]. Several miRNAs have been implicated in controlling insulin signaling and glucose metabolism at multiple levels [15–17]. For example, miR-103 and miR-107 were shown to be up-regulated in the liver of obese mice, and miR-103/107 inhibition increases expression of caveolin-1, a scaffold protein required for caveolae formation, and enhances insulin signaling by increasing insulin receptor stability in the cell membrane [15]. miR-33a and miR-33b were shown to influence insulin signaling and glucose regulation by targeting IRS-2, sirtuin-6, and adenosine monophosphate-activated protein kinase-α1 [16]. Kornfeld et al. performed miRNA microarrays to identify miRNAs deregulated due to obesity and insulin resistance. They used RNA isolated from the livers of high-fat diet-fed mice and db/db mice. They showed that obesity-induced miR-802 impairs glucose metabolism through silencing hepatocyte nuclear factor 1 homeobox B [17].

In this study, we focused on miR-222 because its expression was elevated in both of the aforementioned obese mouse models [17]. Increased miR-222 expression was confirmed in the livers of high fat/high sucrose diet (G+HFHSD)-fed mice, and miR-222 overexpression in hepatocytes caused a substantial decrease in IRS-1 expression, leading to impaired insulin signaling. Furthermore, we demonstrated that miR-222 directly targets the 3′ UTR of *IRS-1* mRNA. Together, these findings suggested a novel mechanism in which up-regulation of miR-222 expression in obesity causes insulin resistance via hepatic IRS-1 repression.

## Materials and methods

### Animals and treatment

Male C57BL/6 mice were purchased from CLEA Japan (Tokyo, Japan). The mice were kept in a temperature-controlled (22±2°C) facility with a 12:12-h light–dark cycle and were given access to food and water *ad libitum*. At 6 weeks of age, the mice were fed normal chow (NC, 3.45 kcal/g, 12.0% fat, 59.1% carbohydrate, and 28.9% protein by calories; CLEA Japan) or HFHSD [4.81 kcal/g, 54.5% fat, 28.3% carbohydrate (including 16.6% sucrose), and 17.2% protein by calories; Oriental Yeast, Tokyo, Japan] for 24 weeks. Furthermore, 6-week-old HFHSD-fed mice were intraperitoneally injected with 0.2 mg/g body weight gold thioglucose (GTG, Wako Pure Chemical Industries, Osaka, Japan) dissolved in phosphate buffered saline (vehicle), which is known to cause hypothalamic lesions and induce hyperphagia followed by obesity and insulin resistance [18]. NC-fed mice were injected with phosphate buffered saline. Body weight was measured every week. Fasting blood glucose and insulin levels were measured every 4 weeks. All mice were killed and livers were collected and frozen in liquid nitrogen for further analysis. Isoflurane and pentobarbital were used for anesthetizing the mice. All procedures were approved by the Animal Care and Use Committee of Kumamoto University (permission number: B27-057).

### Cell culture and transfection of miRNA mimic

Primary hepatocytes were isolated from the livers of male C57BL/6J mice (8–12 weeks old) using the collagenase method as previously described [19]. Each liver was perfused with a perfusion buffer after cannulation of the abdominal inferior vena cava. The hepatic portal vein was cut, and the thoracic inferior vena cava was occluded with forceps. The liver was washed with the perfusion buffer for 3 min and then perfused with a collagenase buffer (0.3 mg/ml) for 13 min. A perfusion rate of 6 ml/min and a temperature around 37°C was maintained for both perfusates during the entire procedure. Hepatocytes were washed and released in cold Dulbecco's Modified Eagle's medium. The cells were separated from undigested tissue with a sterile 70-μm mesh nylon filter. The cell suspension was centrifuged using Percoll (Sigma-Aldrich, St. Louis, MO, USA) to remove dead cells, contaminants, and debris. Hepatocytes were plated on six-well dishes at 0.5 × 10^6^ cells per well and incubated for 12 h in Dulbecco's Modified Eagle's medium containing 10% fetal bovine serum and 1% penicillin–streptomycin. The human hepatocellular carcinoma cell line HuH-7 was provided by RIKEN BRC (RIKEN cell bank, Tsukuba, Japan). HuH-7 cells were incubated in Roswell Park Memorial Institute 1640 medium containing 10% fetal bovine serum and 1% penicillin–streptomycin. Primary hepatocytes and HuH-7 cells were transfected with 30 nM of the miR-222 mimic (product ID: MC11376, Invitrogen, Waltham, MA, USA) or negative control oligos (Invitrogen) using HilyMax (Dojido, Kumamoto, Japan). Cells cultured in serum-free medium overnight were treated with 100 nM insulin for 10 min to examine the expression of gluconeogenic genes and target proteins.

### RNA preparation and quantitative real-time PCR

Total RNA was isolated from tissues or cells using TRIzol (Invitrogen) and reverse-transcribed into cDNA using ReverTraAce-alpha (TOYOBO, Osaka, Japan) as described previously [20]. To assess miRNA expression levels, total RNA was reverse-transcribed using a miR-222 or 221-specific stem-loop primer (Assay ID: 002276, 000524, Applied Biosystems, Waltham, MA, USA) and the TaqMan MicroRNA Reverse Transcription Kit (Applied Biosystems). Quantitative real-time PCR (qRT-PCR) was performed using the LightCycler System (Roche Molecular Biochemicals, Mannheim, Germany) with TaqMan Universal PCR Master Mix II (Applied Biosystems). mRNA, miR-222 and miR-221 transcriptional levels were normalized to 18S, snoRNA202 (Assay ID: 001232, Applied Biosystems) for mice and RNU6B (Assay ID:001093, Applied Biosystems) for human, respectively. Primer sequences used for qRT-PCR are listed in S1 Table.

### Antibodies and immunoblotting

Western blotting (WB) was performed according to standard protocols using antibodies purchased from Cell Signaling Technology (Danvers, MA, USA) [21]. Target proteins were normalized to β-actin. Primary antibodies used for WB are listed in S2 Table. The intensity of the bands was quantified using ImageJ software (National Institutes of Health, Bethesda, MD, USA).

### Dual-luciferase reporter assays

*IRS-1* luciferase reporter constructs [*IRS-1* wild-type (WT)] were constructed by inserting a mouse or human *IRS-1* 3' UTR fragment containing the miR-222 binding site into the pmirGLO Dual-Luciferase miRNA target expression vector (Promega, Madison, WI, USA). This vector is based on *firefly* luciferase (Luc) used as the primary reporter to monitor miRNA regulation with *renilla* luciferase (Rluc) acting as a control reporter for normalization. Luciferase reporter constructs containing the mutated miR-222 binding site were generated by mutation of the mouse or human miR-222 binding site [*IRS-1* mutant-type (Mut)]. Human embryonic kidney (HEK)-293 cells provided by RIKEN BRC were transfected with the luciferase reporter construct together with 30 nM of the miR-222 mimic or negative control oligos using HilyMax. Cells were collected 2 days after transfection and assayed using the Dual-Luciferase Reporter Assay System (Promega).

### Data analysis

All data are presented as mean ± standard deviation (SD) and were analyzed using Welch’s t-test. P values of <0.05 were considered statistically significant.

## Results

### miR-222 expression levels are up-regulated in the livers of G+HFHSD-fed mice

**Fig 1:**
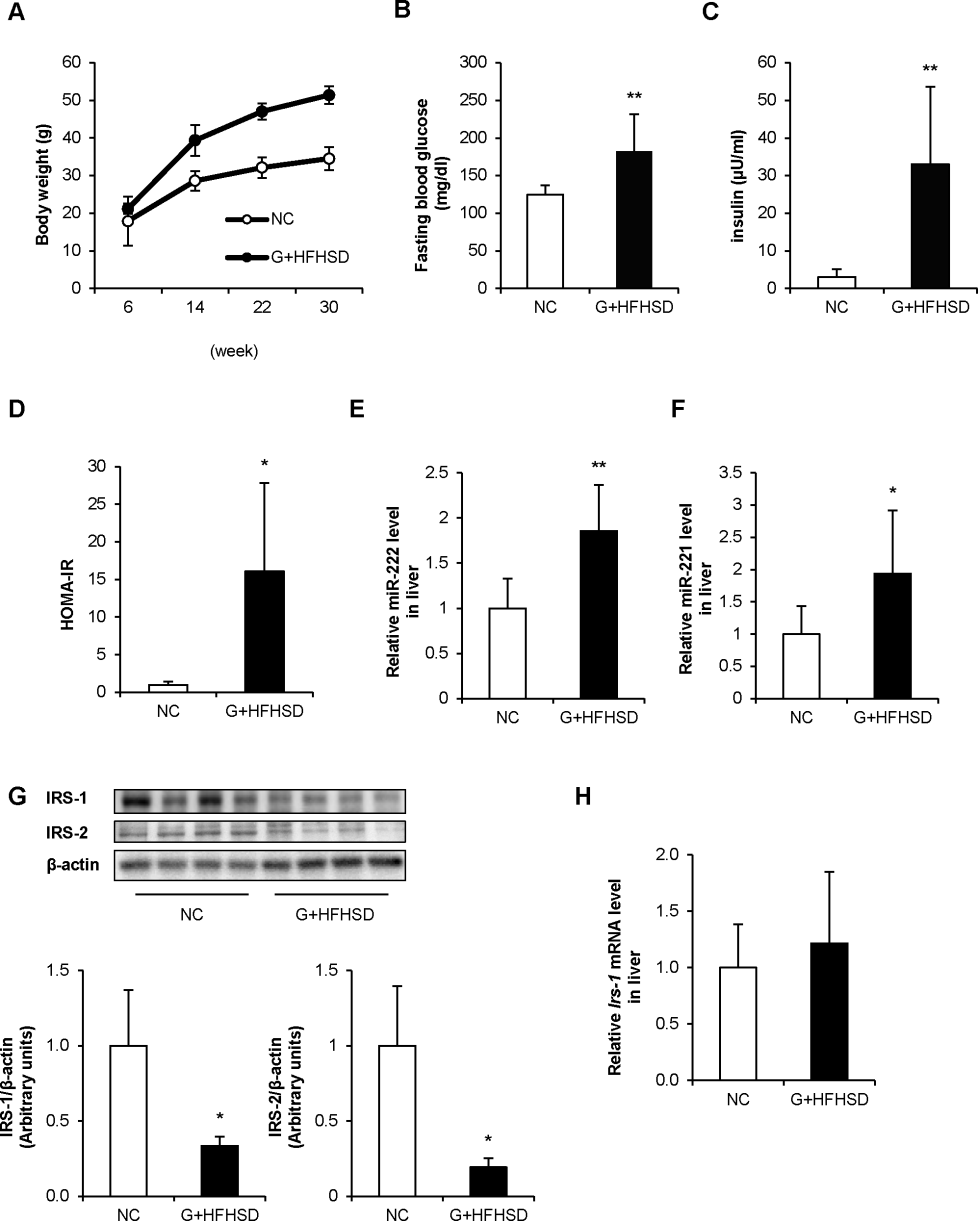
miR-222 expression is up-regulated in the livers of G+HFHSD-fed mice. (A) Body weight was measured during each treatment in NC or G+HFHSD-fed mice (n = 7–8 per group). (B, C) Fasting blood glucose and insulin levels were measured after 24 weeks of treatment. (D) HOMA-IR was calculated using fasting blood glucose and insulin levels after 24 weeks of treatment. (E, F) After 24 weeks of treatment, microRNA was collected from the livers of these mice. miR-222 and miR-221 expression were analyzed by qRT-PCR (n = 8 per group). (G) The proteins in the livers of these mice were analyzed with WB. The IRS-1 and IRS-2 levels were quantified by normalization with β-actin. (n = 4 per group). (H) *Irs-1* mRNA expression in the livers of these mice were analyzed by qRT-PCR. Data are presented as mean ± SD. *p < 0.05, **p < 0.01 compared with NC-fed mice.

C57BL/6 mice were fed NC or HFHSD after GTG injection (G+HFHSD) for 24 weeks. GTG is known to cause hypothalamic lesions and induce hyperphagia followed by obesity and insulin resistance [18]. After 8 weeks from the initiation of HFHSD feeding (at 14 weeks of age), G+HFHSD-fed mice gained more weight compared with NC-fed mice (Fig 1A). However, there were no significant differences in fasting blood glucose and serum insulin levels between the two groups (data not shown). After 24 weeks (at 30 weeks of age), G+HFHSD induced a 48% increase in body weight compared with NC (Fig 1A). Furthermore, fasting blood glucose and serum insulin levels significantly increased in G+HFHSD-fed mice compared with those in NC-fed mice at 24 weeks (Fig 1B and 1C). The homeostasis model assessment of insulin resistance (HOMA-IR, fasting blood glucose [mg/dL] × fasting insulin [μU/mL] /405) was 0.9 versus 16 in NC-fed mice versus G+HFHSD-fed mice at 30 weeks of age (Fig 1D). G+HFHSD-fed mice suffered steatosis, as evidenced by the accumulation of fat droplets in the liver (S1A Fig).

Next, miR-222 expression levels were measured in the livers of these mice. In accordance with previous microarray data [17], miR-222 expression was up-regulated in the livers of G+HFHSD-fed mice (Fig 1E). Furthermore, miR-221, which is a paralog of miR-222, was also up-regulated in the livers of G+HFHSD-fed mice (Fig 1F). On the other hand, up-regulation of miR-222 expression in the skeletal muscle or adipose tissue was not observed (S1B Fig). IRS-1 protein expression but not *Irs-1* mRNA expression was reduced in the livers of G+HFHSD-fed mice (Fig 1G and 1H).

### miR-222 overexpression attenuates insulin-stimulated phosphorylation of Akt and increases in gluconeogenic gene expressions

**Fig 2:**
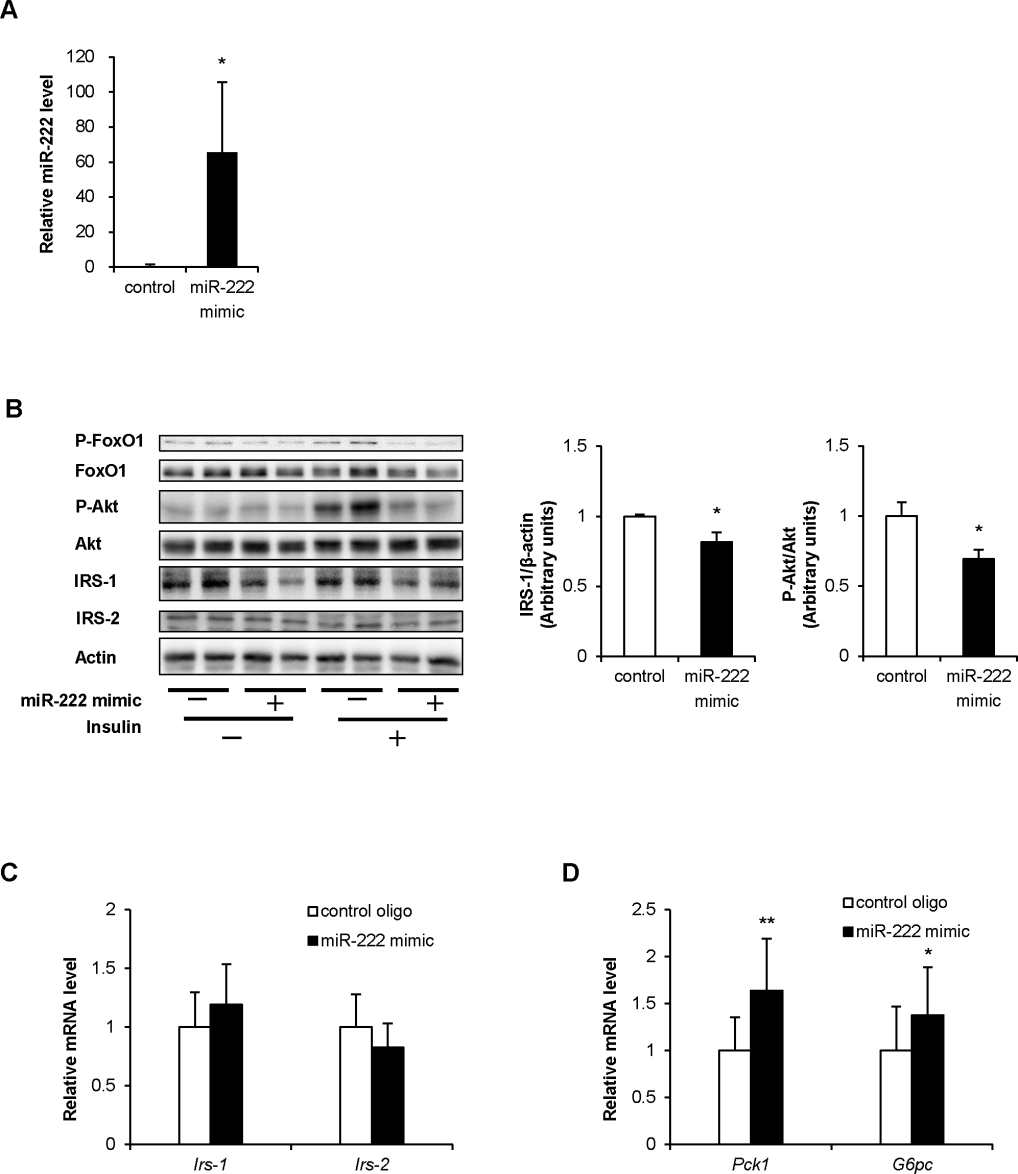
Effect of miR-222 overexpression on insulin signaling in primary mouse hepatocytes. (A) Primary hepatocytes were transfected with 30 nM negative control oligos (control) or miR-222 mimic using HilyMax. After 2 days of transfection, miR-222 overexpression in the cells was confirmed by qRT-PCR (n = 6 per group). (B) Cells overexpressing miR-222 were treated with insulin (100 nM) for 10 min. Cells were harvested, and protein levels involved in insulin signaling were determined by WB. The IRS-1 and P-Akt levels were quantified by normalization with β-actin and total Akt. The values are expressed as mean ± SD from 4 independent experiments. (C) *Irs-1* and *Irs-2* mRNA expression in the cells overexpressing miR-222 were analyzed by qRT-PCR. (D) Each mRNA involved in insulin signaling was analyzed in the cells overexpressing miR-222 (n = 10 per group). Data are presented as means ± SD. *p < 0.05, **p < 0.01 compared with the control group.

To address whether increased miR-222 expression contributed to the development of insulin resistance, we overexpressed miR-222 in primary mouse hepatocytes (Fig 2A). miR-222 overexpression led to a reduction in IRS-1 protein, and insulin-stimulated Akt and FoxO1 phosphorylation (Fig 2B). *Irs-1* mRNA expression was not reduced in the cells overexpressing miR-222 (Fig 2C). In accordance with reduced Akt phosphorylation, phosphoenolpyruvate carboxykinase 1 (*Pck1*) and glucose-6-phosphatase catalytic subunit (*G6pc*), gluconeogenic genes, mRNA abundance under insulin stimulation were increased in the cells overexpressing miR-222 (Fig 2D). These findings suggested that up-regulation of miR-222 expression could affect the development of insulin resistance in the liver.

### Mouse Irs-1 mRNA is a direct target of miR-222

**Fig 3:**
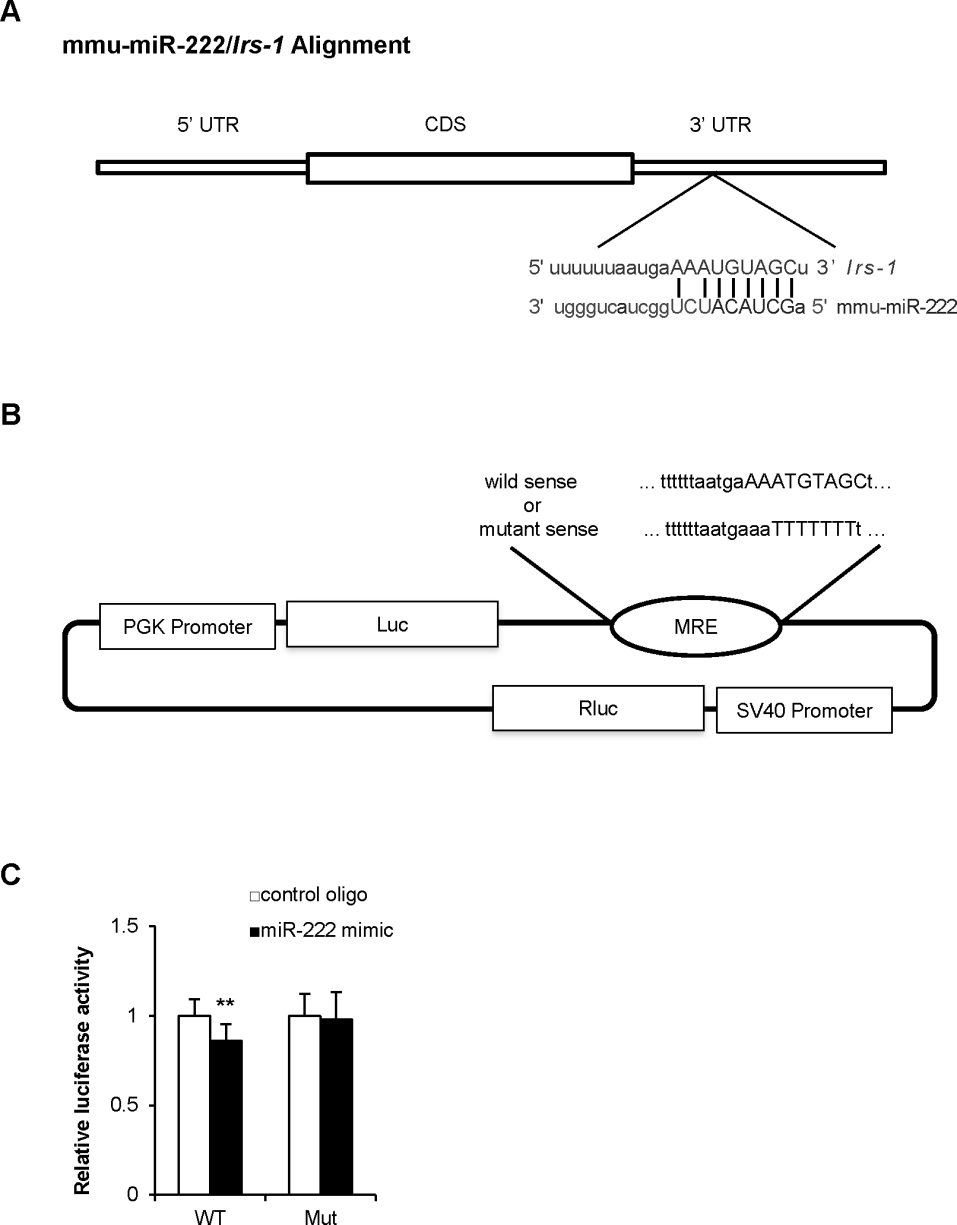
Target site of miR-222 in the 3' UTR of mouse *Irs-1* and assessment of its binding. (A) The seed sequence of miR-222 and the sequence of 3' UTR of *Irs*-*1*. (B) A pmirGLO-based 3′ UTR reporter vector consisting of luciferase cDNA followed by the 3' UTR of murine *Irs-1* mRNA (WT or Mut). (C) HEK-293 cells were co-transfected with the luciferase reporter construct containing WT or Mut 3' UTR of mouse *Irs-1* and the miR-222 mimic or the negative control oligos. After 2 days of treatment, a dual-luciferase assay of these cells was measured (n = 6 per group). Data are presented as mean ± SD. *p < 0.05, **p < 0.01 compared with cells transfected with the negative control group. MRE: microRNA response element; UTR: untranslated region; CDS: coding sequence.

To identify target genes of miR-222, we used miRWalk, which allowed us to aggregate and compare results from other miRNA-to-mRNA databases [22]. Consequently, mouse *Irs-1* was identified as a possible target of miR-222. In fact, IRS-1 protein but not mRNA was decreased in the livers of G+HFHSD-fed mice (Fig 1G and 1H). In hepatocytes overexpressing miR-222, IRS-1 protein expression but not *Irs-1* mRNA expression significantly decreased (Fig 2B and 2C). The expression of IRS-2 is another key insulin-signaling molecule in the liver, but *IRS-2* mRNA does not have any predicted binding site for miR-222 (data not shown). IRS-2 protein expression was also decreased in the livers of G+HFHSD-fed mice but not in the hepatocytes overexpressing miR-222 (Fig 2B and 2C). Fig 3A shows a predicted binding site for miR-222 in the 3′ UTR of mouse *Irs-1* mRNA. To further confirm the direct interaction between miR-222 and the 3′ UTR of *Irs-1* mRNA, a dual-luciferase reporter assay was performed. A pmirGLO-based 3′ UTR reporter vector consisting of luciferase cDNA followed by the 3′ UTR of mouse *Irs-1*, which contained a potential miR-222 binding site (WT), was constructed (Fig 3B). The plasmid, which contained the mutated (Mut) miR-222 binding site, was also analyzed and served as a negative control (Fig 3B). The pmirGLO-based *Irs-1* 3′ UTR reporter was co-transfected with the miR-222 mimic or negative control oligos into HEK-293 cells. miR-222 overexpression significantly inhibited the luciferase activity of the WT *Irs-1* 3' UTR reporter, but it had no effect on the activity of the mutated *Irs-1* 3' UTR reporter (Fig 3C). These results indicated that mouse *Irs-1* mRNA is a direct target of miR-222.

### miR-222 also targets human IRS-1 mRNA

**Fig 4:**
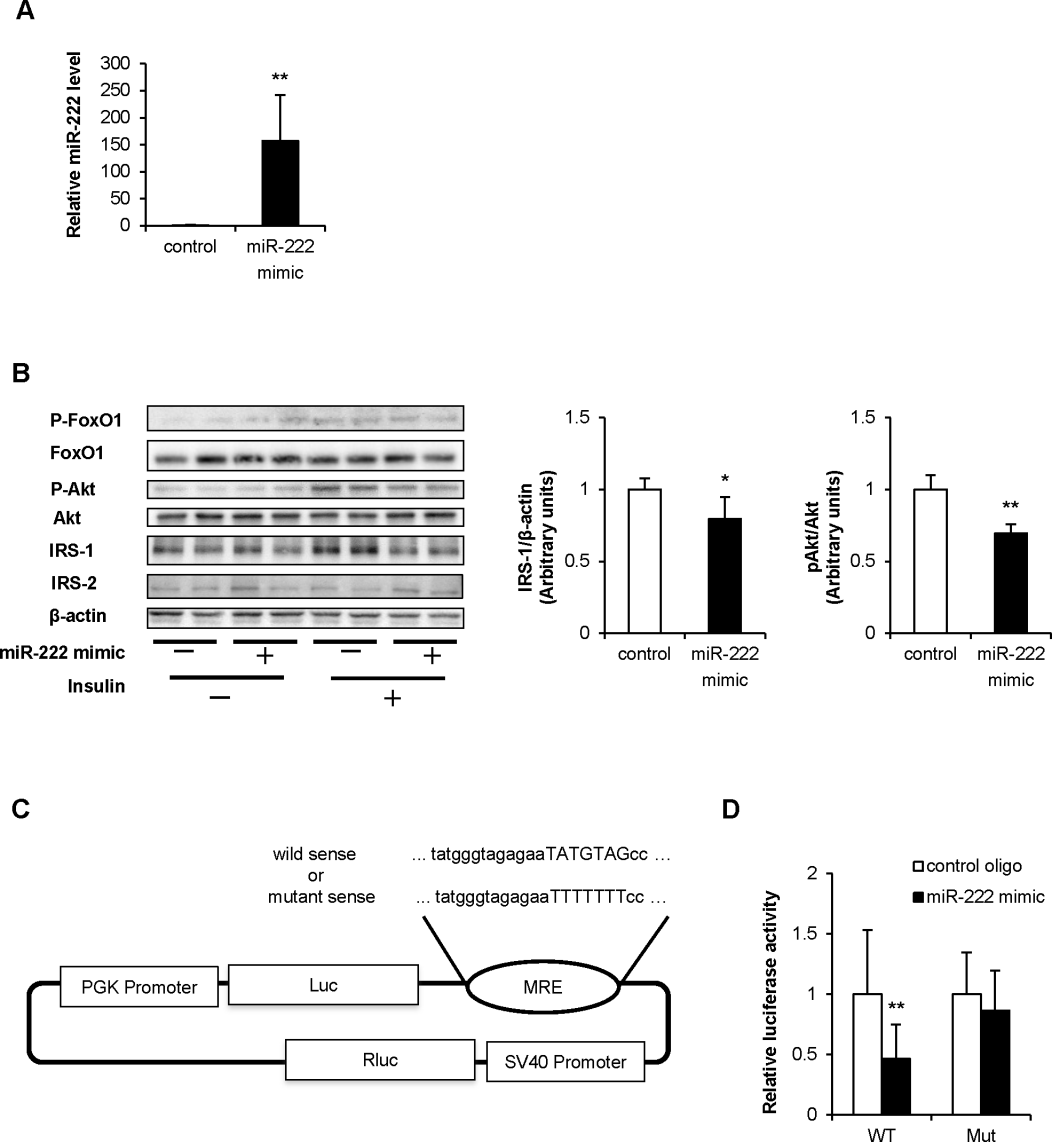
Effect of miR-222 overexpression in HuH-7 cells. (A) HuH-7 cells were transfected with 30 nM of the negative control or miR-222 mimic. After 2 days of transfection, miR-222 overexpression was confirmed by qRT-PCR (n = 6 per group). (B) Cells overexpressing miR-222 were treated with insulin (100 nM) for 10 min. Cells were harvested, and protein levels involved in insulin signaling were determined with WB. The IRS-1 and P-Akt levels were quantified by normalization with β-actin and total Akt. The values are expressed as the mean ± SD from 4 independent experiments. (C) The pmirGLO-based 3' UTR reporter vector consisted of luciferase cDNA followed by the 3' UTR of the WT or Mut human *IRS-1* mRNA. (D) HEK-293 cells were co-transfected with the luciferase reporter construct containing WT or Mut 3' UTR of human *IRS-1* and the miR-222 mimic or the negative control oligos. After 2 days of treatment, a dual-luciferase assay of these cells was measured (n = 10 per group). Data are presented as mean ± SD. *p < 0.05, **p < 0.01 compared with the control group.

The 3' UTR of human *IRS-1* mRNA was also a predicted binding site for miR-222. Therefore, we performed similar experiments using HuH-7 cells, a human hepatoma cell line. In HuH-7 cells overexpressing miR-222 (Fig 4A), IRS-1 protein expression, and insulin-induced Akt and FoxO1 phosphorylation were significantly decreased (Fig 4B). The pmirGLO-based human *IRS-1* 3' UTR reporter vector, containing a potential miR-222 binding site (WT) or a mutated miR-222 binding site (Mut), was constructed (Fig 4C). miR-222 overexpression decreased the luciferase activity of the WT human *IRS-1* 3' UTR reporter, but had no effect on the mutated human *IRS-1* 3' UTR reporter (Fig 4D). Therefore, *IRS-1* is most likely a target gene of miR-222 in humans as well.

## Discussion

In this study, we demonstrated that miR-222 expression is up-regulated in the livers of G+HFHSD-fed mice and further showed that *Irs-1* mRNA is a target of miR-222. Previously, miR-222 was found to play a role in cancer cell growth and cell cycle progression via directly targeting p27, p57, and PTEN [23]. In addition, metformin, which is an oral antidiabetic drug administered to improve insulin sensitivity, reduced miR-222 expression in cancer cell lines such as A549 and NCI-H358 human lung cancer cell lines. Coleman et al. reported increased miR-221/222 expression in the internal mammary arteries of patients with diabetes. However, miR-221/222 expression levels in patients with diabetes treated with metformin were comparable to those of individuals without diabetes [24]. They showed a significant inverse correlation between the dose of metformin and the levels of miR-221/222. These results indicated a strong relationship between insulin resistance and miR-222 levels, but the exact roles of miR-222 in insulin signaling had yet to be defined.

As previously mentioned, our study showed up-regulation of miR-222 expression in the livers of G+HFHSD-fed mice. We did not observe miR-222 up-regulation in the skeletal muscle or adipose tissue of these mice. A recent study of circulating miRNA profiling demonstrated an increase in miR-222 expression in the plasma of patients with type 2 diabetes [25]. To note, circulating miR-222 was markedly decreased upon metformin treatment. On the other hand, it was increased by intralipid infusion. Li et al. reported that high glucose stimulation increased miR-221 expression in human umbilical vein endothelial cells [26]. We stimulated primary hepatocytes with high glucose or a high dose of insulin, but we did not observe a change in miR-222 levels (data not shown). It has been reported that some adipokines or interleukins such as tumor necrosis factor–α, interferon–γ [27], or other hormones such as estradiol [28], which are dysregulated in insulin resistant states affect miR-222 expression. Further studies are needed to identify factors that increase miR-222 levels in liver under the state of insulin resistance.

We also demonstrated that miR-221, which is a paralog of miR-222, was up-regulated in the livers of G+HFHSD-fed mice. Because the seed sequences of miR-222 and miR-221 are identical, both may be able to affect the same target genes. Furthermore, it has been reported that these two miRNAs have similar functions and expression patterns [29,30]. However, miRNAs with the same seed sequences do not always regulate the same target genes [31]. Further examination is needed to demonstrate the role of miR-221 in future experiments.

To address whether increased miR-222 expression contributed to the development of insulin resistance, we overexpressed miR-222 in primary mouse hepatocytes. In these cells, insulin-stimulated Akt phosphorylation dramatically decreased. In accordance with reduced Akt phosphorylation, *Pck1* and *G6pc* mRNA expressions were elevated. In addition, we showed that *Irs-1* mRNA is a target of miR-222. Both mouse and human *IRS-1* 3′ UTRs contain a predicted binding site for miR-222, and IRS-1 protein expression was reduced in hepatocytes and HuH-7 cells overexpressing miR-222. Furthermore, we confirmed that the 3′ UTRs of both mouse and human *IRS-1* mRNAs are indeed direct targets of miR-222. In our present experiments, the decrease in IRS-1 protein by miR-222 overexpression was only 20% in primary hepatocytes. However, in the liver of G+HFHSD-fed mice, 2-fold increase of miR-222 led to a 60% reduction in IRS-1 protein. *In vivo*, various factors can affect insulin signaling, especially under insulin resistant states. For example, some adipokines and interleukins are dysregulated in insulin resistant states and known to impair insulin signaling at the multiple levels of downstream molecules. We consider up-regulation of miR-222 could be one of the factors that represses IRS-1 expression in the livers of obese insulin resistant models. Thus far, miR-29a, miR-126, miR-144, and miR-96 have been reported to repress IRS-1 expression and impair insulin signaling [32–34]. IRS-1 protein expression levels are known to be lower in the liver and muscle of ob/ob mice [35]. Actually, IRS-1 protein expression was decreased in the skeletal muscles and adipose tissues of our mouse models. However, miR-222 expression was not increased in these mouse models. Therefore, in our present experiments, miR-222 induced reduction in IRS-1 levels was confirmed only in the livers but not in the muscles or adipose tissues.

We also confirmed reduced IRS-1 protein but not *Irs-1* mRNA expression in the liver of G+HFHSD-fed mice. Previously, we also reported that IRS-1 protein but not mRNA expression or promoter activity decreased due to long-term exposure to insulin in 3T3-F442A adipocytes [3]. We suspect that post-transcriptional regulation by miRNA might be one of the reasons for differences between *Irs-1* mRNA and protein expression profiles under insulin-resistant states. Furthermore, IRS-2 protein expression was decreased in the liver of G+HFHSD-fed mice. *In vivo*, the impairment of insulin signaling in the liver under insulin-resistant states must be due to the decrease of not only IRS-1 but also IRS-2 expression. *In vitro*, however, miR-222 overexpression did not alter IRS-2 protein expression, and *IRS-2* mRNA did not have any predicted binding site for miR-222. Hence, we concluded that miR-222 could involve hepatic insulin resistance at least in part through IRS-1 repression.

In summary, we have demonstrated that hepatic miR-222 is up-regulated in an insulin-resistant state, which in turn impairs insulin signaling through the repression of IRS-1 expression. These findings suggest that miR-222 could be a novel target for the treatment of obesity-associated metabolic disorders.

## Supporting information

S1 Fig (A) A representative section stained with hematoxylin/eosin in the livers of NC or G+HFHSD-fed mice. (B) microRNA was collected from the quadriceps femoris muscle and epididymal adipose tissue of NC or G+HFHSD-fed mice.
(TIF)

S1 Table Primer sequences used for qRT-PCR. (TIF)

S2 Table Primary antibodies used for Western blotting. (TIF)
