# Supplementary figures and images for: Identification of microRNA that represses IRS-1 expression in liver
Source: PLoS One. 2018 Jan 24;13(1):e0191553. doi: 10.1371/journal.pone.0191553 (PMC5783395; doi:10.1371/journal.pone.0191553)

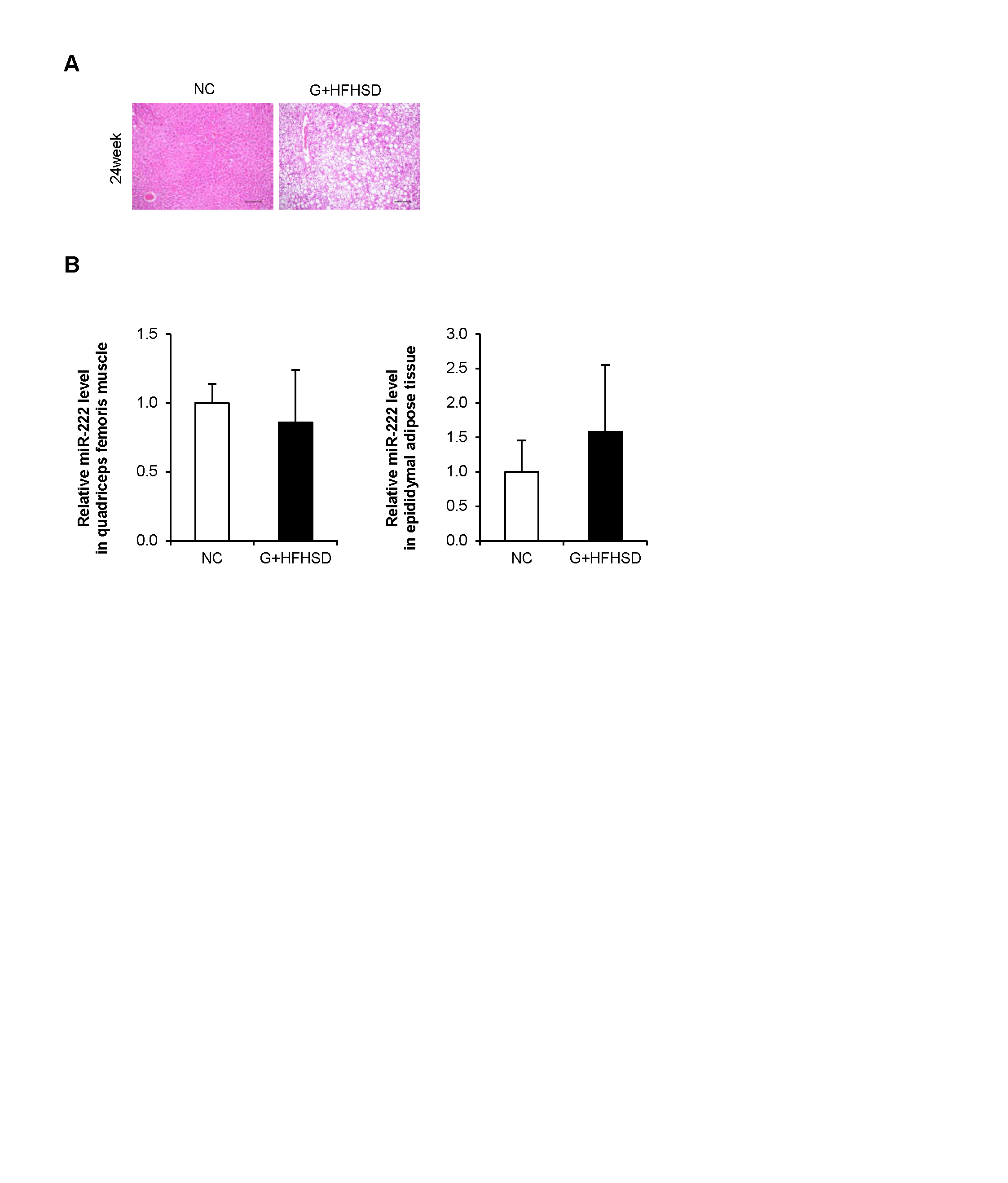

Supplement: S1 Fig — (A) A representative section stained with hematoxylin/eosin in the livers of NC or G+HFHSD-fed mice. (B) microRNA was collected from the quadriceps femoris muscle and epididymal adipose tissue of NC or G+HFHSD-fed mice. (TIF) [file pone.0191553.s001.tif]
